# Real-World Data on Antibiotic Group Treatment in European Livestock: Drivers, Conditions, and Alternatives

**DOI:** 10.3390/antibiotics11081046

**Published:** 2022-08-03

**Authors:** Julia Jerab, Wiebke Jansen, John Blackwell, Jobke van Hout, Andreas Palzer, Stephen Lister, Ilias Chantziaras, Jeroen Dewulf, Nancy De Briyne

**Affiliations:** 1 Federation of Veterinarians of Europe (FVE), Rue Victor Oudart 7, 1030 Brussels, Belgium; info@fve.org (J.J.); wiebke@fve.org (W.J.); 2 Brownlow Veterinary Group Ltd., Ellesmere Business Park, Oswestry Rd., Ellesmere SY12 0EW, UK; johnmblackwell@btinternet.com; 3 Royal GD, Arnsbergstraat 7, 7418 EZ Deventer, The Netherlands; j.v.hout@gddiergezondheid.nl; 4 Veterinary Pig Practice Scheidegg, Bahnhofstrasse 30, D-88175 Scheidegg, Germany; a.palzer@lmu.de; 5 Lister Veterinary Consultancy, 125 The Street, Norwich NR8 5DF, UK; salister@crowshall.co.uk; 6 Veterinary Epidemiology Unit, Faculty of Veterinary Medicine, Ghent University, Salisburylaan 133, 9820 Merelbeke, Belgium; ilias.chantziaras@ugent.be (I.C.); jeroen.dewulf@ugent.be (J.D.)

**Keywords:** antibiotics, metaphylaxis, antimicrobial resistance, alternative therapy options

## Abstract

Major efforts have been made by veterinary professionals to reduce the need for antibiotic use in animals. An online survey launched by the Federation of Veterinarians of Europe (FVE) aimed to gather responses from practicing veterinarians with field experience in metaphylactic livestock group treatment. Only 17% of all veterinarians (n = 183/1087, all species-specific responses merged) applied metaphylactic group treatments to 75% or more of all their treatments. Significantly less metaphylactic group treatments were reported in mixed practices (*p* = 0.002) and practices specialized in cattle (*p* < 0.001) as well as small (*p* = 0.007) and very small practices (*p* = 0.009). Gram-negative bacteria, mostly composed of *Enterobacteriaceae* and *Pasteurellaceae*, were considered by 75.3% (n = 967/1385) as the most devastating bacterial pathogens. Respondents alleged morbidity (20.1%, n = 201/998) and mortality (42.2%, n = 421/998) as major consequences for animal health and welfare if metaphylaxis would be banned. Responding veterinarians pointed towards vaccinations; improved biosecurity, including hygiene measures; and improved herd health management as the three most effective alternative measures to prevent metaphylactic treatment. However, more research is needed on how to implement appropriate alternatives in a holistic hurdle approach. Active support on a national level will be necessary for the development and application of targeted veterinary treatment guidelines for practitioners, which promote the understanding of drivers and include initiation criteria for metaphylactic group treatments in livestock.

## 1. Introduction

Major efforts have been made by the veterinary professionals and auxiliary animal health professionals to reduce the need for antibiotic use in farmed animals, resulting in a 43.2% decrease of antimicrobial veterinary products sales in the European Union (EU) between 2011 and 2020 [1]. However, animals can become sick even under the best rearing conditions and may need to be treated with antibiotics [2]. The term group treatment encompasses both prophylaxis and metaphylaxis. Prophylaxis is defined as the administration of a veterinary medicinal product (VMP) to an animal or group of animals before clinical signs of a disease, to prevent the occurrence of disease or infection [3]. In contrast, metaphylaxis is defined as the administration of a VMP to a group of animals after a diagnosis of clinical disease in part of the group has been established, with the aim of treating the clinically sick animals and controlling the spread of the disease to animals in close contact and at-risk and which may already be subclinically infected [4]. The new Regulation (EU) 2019/6 states that ‘Antimicrobial medicinal products shall be used for metaphylaxis only when the risk of spread of an infection or of an infectious disease in the group of animals is high and when no other appropriate alternatives are available’ [5].

Today’s livestock husbandry practices include the rearing of animals for food production in groups of similar-aged individuals, often stemming from different litters on farm or even origins. Though all major farmed animals are gregarious, weaning represents a major stressor and subsequent (intra- or inter-farm) transport followed by regrouping adds to this, especially for calves. Some studies demonstrated that metaphylaxis in commercially reared cattle and pig holdings was most often implemented after weaning, transportation, and co-mingling, as these periods of stress are often followed by disease [6,7,8]. The antibiotic sales data mirror these findings as they represent a surrogate measure for animal group medications. The European Surveillance of Veterinary Antimicrobial Consumption (ESVAC) 2021 report revealed that 86.9% of the antimicrobial products sold for veterinary care in Europe were products suitable for group treatment i.e., oral powders, oral solutions, and premixes [1]. While ESVAC does not classify injectable antibiotics as suitable for group treatment, long-acting injectable antibiotics can also be used for metaphylaxis, particularly for suckling piglets and veal calves.

Overall, there is a lack of quantitative real-world data on objectives, drivers, most common conditions, and specific criteria on when and how metaphylaxis is applied in European livestock (referring in this paper to cattle, small ruminants, pigs, lagomorpha and poultry) as well as alternatives thereof. Metaphylaxis is initiated typically when several animals within a group display clinical manifestation and subsequent diagnosis of an infectious disease. European national action plans, such as the Belgian action plan, stipulated by the Belgian knowledge centre on antibiotic use and resistance in animals (AMCRA), already guide the decision to initiate metaphylactic treatment based on different criteria and knowledge of the type/s of pathogens involved or results of antimicrobial susceptibility testing (AST) [9]. While studies demonstrated metaphylaxis to be a successful method for reducing morbidity and mortality, group treatment of animals with antibiotics also harbors risks. Antibiotic mis- and overuse in animals were identified as the main driving forces behind the development of antimicrobial resistance (AMR) in bacteria [10,11]. Metaphylactic treatment with antibiotics has a significant impact on the development of AMR [12,13]. Checkley et al. (2010) found an association between the use of oxytetracycline, either in feed or as an injectable, with the development of resistance in fecal *Escherichia coli* in feedlot cattle [12]. Mazurek et al. (2015) also used commensal *E. coli* as an indicator bacterium for AMR development and found a strong association between resistance and metaphylactic trimethoprim and sulfamethoxazole use in pigs [13]. Therefore, antibiotic stewardship, and measures to reduce the need to treat farmed animals with antibiotics aim at improved animal welfare, nutrition, genetics, and better use of biosecurity, including hygiene measures and vaccination schemes [14,15,16]. Moreover, coaching strategies such as guided interventions as a joint effort of pig farmers and their herd veterinarian/other advisors have shown to be a promising tool in the reduction of antimicrobial use (AMU) [17]. Research into alternatives to antibiotics is also well underway, including pre- and probiotics and bacteriophages [18,19,20,21]. Nevertheless, metaphylactic treatment with antibiotics is considered to be an indispensable tool for veterinarians under certain conditions. The aim of this survey was to collect representative information from veterinary practice on drivers and most common settings of metaphylaxis in livestock, including bacterial species and clinical conditions per animal species at different production stages. In addition, consequences on animal health and welfare as well as most promising alternative therapy options for metaphylactic treatment were explored.

## 2. Results

A total of 714 responses were received, of which 662 responses met the inclusion criteria (i.e., veterinarians working with terrestrial livestock and/or poultry). Geographically, most responding veterinarians were working in Spain (n = 227, 34.3%), followed by France (n = 108, 16.3%), Germany (n = 61, 9.2%), Hungary (n = 46, 7%), the Netherlands (n = 42, 6.3%), and Poland (n = 35, 5.3%). The majority of responding veterinarians worked in a mixed practice (n = 241, 36.4%), followed by veterinarians working in practices specialized in cattle (n = 166, 25.1%) and pigs (n = 141, 21.3%). Most had ≥25 years of experience (n = 271, 41%) and only a minority of the responding veterinarians had ≤5 years of experience (n = 54, 8.2%), while the number of veterinarians with 6–15 years of experience (n = 178, 24%) was relatively similar to those with 16–25 years of experience (n = 159, 26.9%). Veterinary practices were categorized into four sizes: very small with 1–3 veterinarians working in the practice, small with 4–6 veterinarians, medium with 7–9 veterinarians, and large with 10 or more veterinarians. With the exception of practices specialized in cuniculture, most responding veterinarians worked in very small practices (n = 332, 49%). Practices specialized in cuniculture were mostly small practices (n = 4, 50%). Table S1 details the demographic features and Table S2 the complete questionnaire (Q1–Q9).

### Metaphylactic Treatment Conditions

#### Percentage of Metaphylactic or Group Treatments

**Figure 1:**
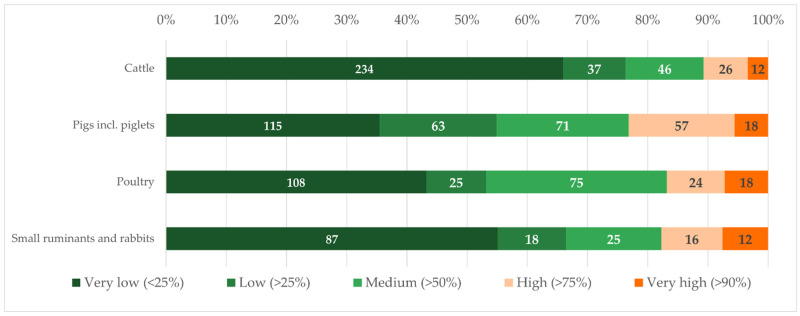
Quantitative metaphylaxis use per species in absolute numbers (inside the bars) and in per cent (x-axis) based on n = 1087 survey responses to Q6 (multiple answers possible, all species-specific responses merged) in six categories: Very low (<25%), Low (>25%), Medium (>50%), High (>75%), Very high (>90%).

Veterinarians indicating that more than 75% of their treatments were metaphylaxis, did this significantly less often (*p* < 0.001) for cattle than for other livestock (Figure 1). The logistic regression model confirmed this, as veterinarians who indicated that metaphylaxis made up more than 75% of their treatments, worked significantly less often in mixed practices (*p* = 0.00165) and practices specialized in cattle (*p* < 0.001), as well as in small (*p* = 0.007) and very small practices (*p* = 0.009). The logistic regression showed neither that the country of residence nor the experience significantly influenced the use of metaphylaxis of responding veterinarians.

Veterinarians, asked when they make the decision to apply metaphylactic treatment, replied that it was equally dependent on the severity of disease, the capacity of the disease to spread, and further laboratory testing. No significant differences were detected in the decision-making to initiate metaphylactic treatment per practice type (Table 1).

**Figure 2:**
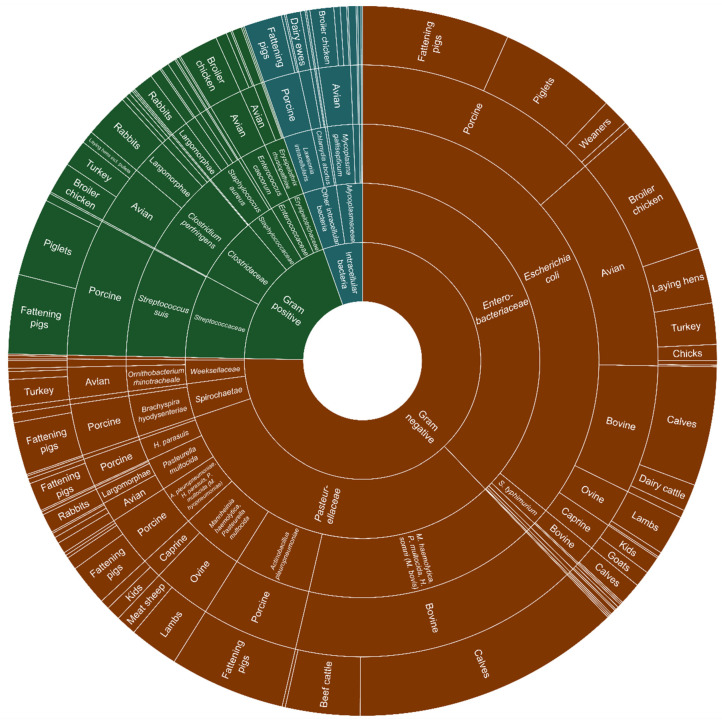
Sunburst chart plotted in Excel showing gram-negative bacteria (brown), gram-positive bacteria (green) and intracellular bacteria (blue) and the total relative abundance of bacteria classification (first two interior circles), bacteria species (third circle), affected animal species (fourth circle) and production stage (exterior circle) indicated by responding veterinarians (Q8 with n = 1385 responses, multiple answers possible) as causing the infections with the most devastating effect on animal health and welfare.

Gram-negative bacteria accounted for 75.3% of the total of 1385 bacterial pathogens that responding veterinarians indicated (Q8, multiple answers possible, all species-specific responses merged) to have the most devastating effect on animal health and welfare if metaphylaxis were to be banned. *Enterobacteriaceae* (n= 524/1385, 37.8%), and thereof *E. coli* (n = 497/524, 95%), represented the majority of responses in all species and all types as well as various *Pasteurellaceae* (n = 443/1385, 32%). Among the Gram-positive bacteria, the most common were *Streptococcus suis* for pigs (mainly involving piglets and fattening pigs (n = 104/1385, 7.5%) and *Clostridium perfingens* for avian species, including broiler chicken, meat turkeys, and laying hens as well as rabbits (n = 82/1385, 5.9%). Intracellular bacteria were mainly represented by *Mycoplasma* spp. for avian species, and thereof mainly *Mycoplasma gallisepticum* in broiler chicken (n = 24/1385, 1.7%) (Figure 2).

**Figure 3:**
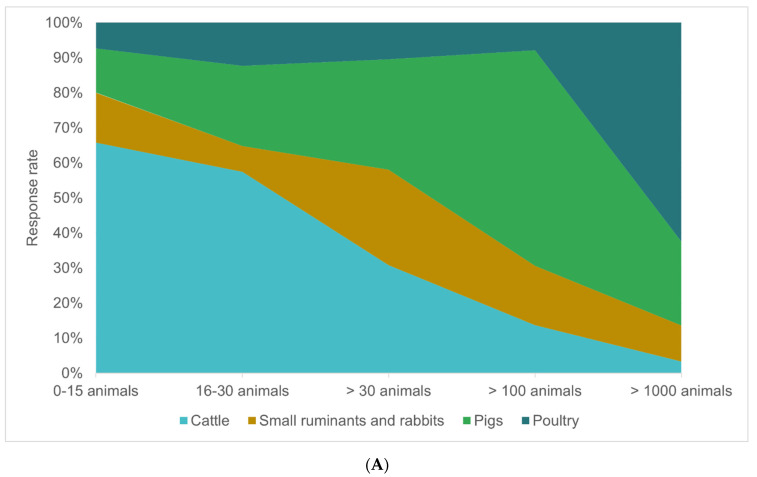

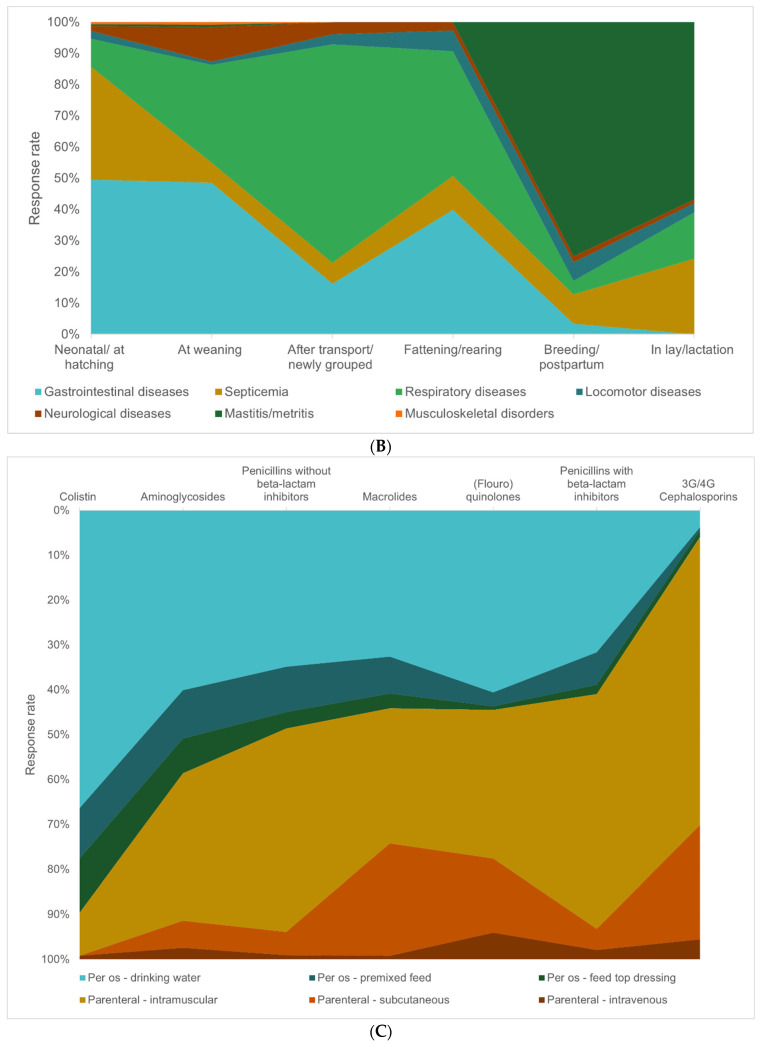
Most frequent models for metaphylactic treatment as indicated by responding veterinarians in percent (x-axis). (**A**): species (cattle, pigs, small ruminants and rabbits, poultry) and group size in five categories (0–15 animals, 16–30 animals, >30 animals, >100 animals, >1000 animals.) (**B**): stage of production (neonatal/ at hatching, at weaning, after transport/ newly grouped, fattening/rearing, breeding/ postpartum, in lay/lactation) and disease (gastrointestinal diseases, septicemia, respiratory diseases, locomotor diseases, neurological diseases, mastitis/metritis, musculoskeletal disorders). (**C**): antibiotic class (colistin, aminoglycosides, penicillins without beta-lactam inhibitors, macrolides, (flouro) quinolones, penicillins with beta-lactam inhibitors, 3G/4G cephalosporins), and route of administration (blue shades: per os-drinking water, per os-premixed feed, per os-feed top dressing, orange shades: parenteral-intramuscular, parenteral-subcutaneous, parenteral-intravenous).

In respect to the most frequent model for metaphylactic treatment that applies per practice, responding veterinarians indicated that for groups of 0–15 individuals, cattle were the most common species receiving metaphylactic treatment (n = 125/190, 65.8%). For group sizes of >100 individuals, pigs were the most common species (n = 149/242, 61.6%), while for group sizes >1000 individuals the species most frequently indicated (n = 133/213, 62.4%) was poultry (Figure 3A). At the neonatal/hatching stage, the most frequent indications for metaphylactic treatment were gastrointestinal diseases (n = 160/323, 49.5%) and septicemia (n = 117/323, 36.2%). Respiratory diseases were increasingly indicated at the weaning stage (n = 117/373, 31.4%) and fattening/rearing stage (n = 103/258, 39.9%) and were the most common indication at the after transport/newly grouped stage (n = 216/309, 69.9%). Mastitis/metritis was the most common indication during the breeding/post-partum stage (n = 153/204, 75%) and lay/lactation stage (n = 96/169, 56.8%) (Figure 3B). Looking at the antimicrobial classes, the majority of colistin was administered per os (n = 216/241, 89.6%), thereof 74.1% being administered through drinking water (n = 160/216). In contrast, third and fourth generation cephalosporins were almost exclusively administered parentally (n = 126/134, 94%) (Figure 3C). Only practices specialized in poultry showed a significant correlation of their metaphylactic use in respect to the application route: With respect to their percentage of metaphylactic treatments, responding poultry practitioners with a high metaphylactic use (>75% of all treatments) administered antibiotics significantly more often (*p* < 0.001) antibiotics per os, and the correlation was specifically evident for fluoroquinolones, macrolides, aminoglycosides, penicillins without beta-lactam inhibitors, and penicillins with beta-lactam inhibitors (each *p* < 0.001, resp.), compared to practitioners with a lower percentage (<75%) of metaphylactic treatment. The same was apparent for practitioners working with small ruminants and lagomorpha (classified as ‘other species’), but solely for aminoglycosides (*p* = 0.028).

Veterinarians from all practice types considered increased mortality (n = 421/998, 42.2%) and increased morbidity (n = 201/998, 20.1%) to be the most significant health consequences if metaphylaxis were to be prohibited (multiple answer possible, all species-specific responses merged). For all practice types, except for poultry, the third most significant consequence according to responding veterinarians was decreased production and economic loss (n = 134/998, 13.4%). For poultry practitioners, the third most significant consequence was lower welfare (n = 34/184, 18.5%) (Table 2).

**Figure 4:**
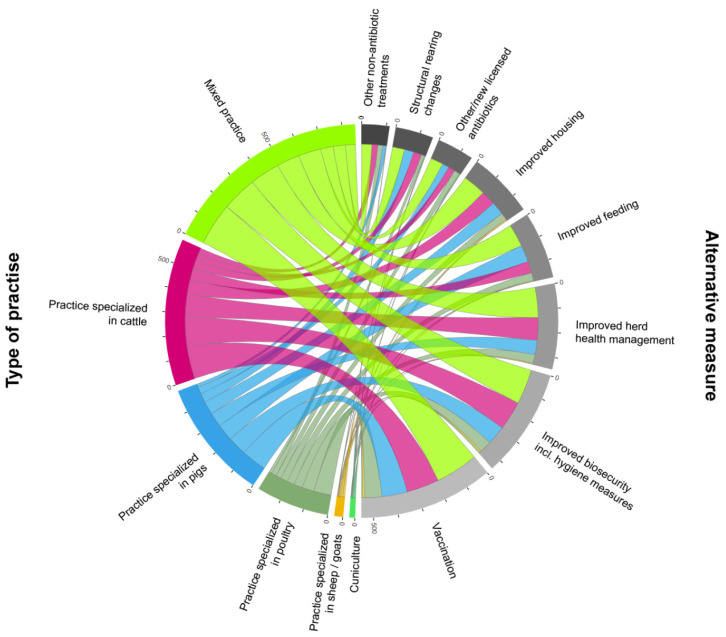
Chord diagram displaying the inter-relationship between most effective alternative measures to prevent and avoid the need for metaphylactic treatment per practice type (Q9 with n = 2329 responses, multiple answers possible). Arc lengths on the outer circle are proportional to total quantities. Plotted with JavaScript in HTML based on an open library from https://d3js.org/ (accessed on 31 March 2022).

Responding veterinarians indicated vaccinations (n = 548/2329, 23.5%) and improved biosecurity including hygiene measures (n = 444/2329, 19.1%) as the two most effective alternative measures to prevent and to avoid the need for metaphylactic treatment (multiple answer possible, all species-specific responses merged). Regulatory changes and alternative and complementary measures (pre-, probiotics, phytotherapy, etc.) were perceived as least effective with 18 and 17 responses and are not shown in Figure 4.

## 3. Discussion

There is an increased public awareness of how livestock is reared for food production, with a special emphasis on animal welfare and antibiotic use. In the last 20 years, veterinary professional associations have been progressively committed to decrease the need for antibiotic use through the promotion of better husbandry, better disease monitoring and surveillance systems and biosecurity measures. The new VMP Regulation (EU) 2019/6 came into force in January 2022 and introduced additional requirements, including the collection of antibiotic use data per species, a list of antimicrobials reserved for human use only, and specific conditions for metaphylactic use of antibiotics [5]. However, even animals that are kept in the best conditions can become sick, and animals deserve treatment, too [2]. This survey gathered 662 responses from veterinary livestock practitioners on the conditions of their metaphylactic treatments.

### 3.1. Use of Metaphylaxis per Species and Practice Size

More than half of the poultry practitioners (n = 133/250, 53.2%) indicated very low to low metaphylaxis use, which is in sharp contrast to current market practices: Firstly, poultry is almost exclusively held in larger flocks and any disease diagnosed in poultry flocks requires treatment of the whole group irrespective of the morbidity rate and therefore inevitably treating a proportion of animals which are not (yet) showing clinical signs, reflecting the legal definition of metaphylaxis [6,8,10]. Secondly, VMPs for poultry are almost exclusively available as oral formulations and exclude therefore individual treatment [3,4]. This discrepancy is potentially due to differing interpretations of legal metaphylactic treatment. It might be that many poultry practitioners extrapolate quickly the severity of diseases based on the gross pathology and their experience, considering the whole flock as diseased and requiring treatment. The European Medicines Agency (EMA) defines five states of diseases through which a host may progress following exposure to a pathogen and according to the host–microbe interaction based on Casadevall and Pirofski (2000) [22]. There is only a thin line between state 2 (colonized, not infected, no disease) to state 3 (infected, no disease) and state 4 (infected, sub-clinical disease) [3]. This thin line between prophylactic and metaphylactic treatment is often blurred under field conditions.

Responding veterinarians working in small and very small practices were less likely to administer metaphylactic treatment. This was potentially due to veterinarians in smaller practices predominantly working with less integrated farms where individual treatment is more feasible and fosters a trusted vet–farmer relationship. This was supported by multiple studies, which showed that veterinarians are considered the primary advisors for farmers [22,23,24,25]. Veterinarians working in smaller practices could have a better relationship with farmers as they are more likely to have repeated visits with the same farmer, thereby establishing a better vet–farmer relationship with stronger communication, compliance, and collaboration [26,27]. The collaboration established due to repeated visits from the same veterinarian was shown to increase the knowledge of farmers on the issue of AMR and improved compliance to advise regarding decreased AMU as strategy to combat AMR [28,29]. Farm size was discussed controversially as a risk factor, some studies showed that larger veal calf and pig farms had a significantly higher treatment frequency with antimicrobials [30,31]. However, other European studies did not find this association [32,33]. Mixed practices and practices specialized in cattle had a significantly lower metaphylactic use when compared to other practice types in our survey. Few veterinarians indicated that they apply metaphylactic treatment in cattle groups with more than 100 individuals (n = 33/279, 11.8%). This suggests that the responding veterinarians mostly treat small herds of calves or individual adult cattle, but work not exclusively with large veal calf holdings, which were shown to administer the majority of antibiotics for metaphylactic or prophylactic treatments (see further) [7]. Thus, this contributed to the lower metaphylactic group treatments in mixed practices and practices specialized in cattle.

The country of residence was not significantly influencing the metaphylactic use, which might be due to the geographical bias, as most responses were received from countries with above average antibiotic sales [1]. However, the ESVAC report indicated that sale differences between countries can be partly explained by differences in animal demographics, occurrence of bacterial diseases, selection of antimicrobial agents, dosage regimes, types of data source, and veterinarians’ prescribing habits [1].

### 3.2. Initiation Reasoning of Metaphylaxis

The survey indicates that the severity of the disease, the perception of capacity of a disease to spread quickly, and further laboratory testing, including AST equally influenced responding veterinarians’ choice to initiate metaphylactic treatment. This is coherent with an earlier survey on factors influencing antibiotic prescribing habits of European veterinarians, in which veterinarians working with food-producing animals indicated AST to be the most influential factor in their prescribing habits [34]. Further laboratory testing such as AST has been integrated into multiple national action plans as a mandatory step before the use of certain critical antibiotics [9,35] and is included in the EU guidelines for national action plans to combat AMR [36]. Alongside national action plans, laboratory testing should be made more routine and accessible by decreasing the time between testing and results and by lowering the price of AST [34]. The spread of disease was a major driver for the initiation of metaphylaxis, acknowledging that the control and prevention of a disease epidemic should be the true purpose of antibiotic metaphylaxis. To control these disease dynamics, multiple intrinsic and extrinsic factors have to be considered, e.g., the quality and quantity of immunological coverage can be diminished by stress factors e.g., (early) weaning, castration, and disbudding [37]. Animal husbandry practices such as high stocking density or poor ventilation favoring the spread of infectious diseases weaken the immune system additionally [38]. Metaphylaxis represents therefore one of the most precious tools in the veterinary toolbox and must be employed with utmost care and consideration, under strict veterinary oversight alongside other improvements of animal husbandry methods. Therefore, a morbidity threshold (e.g., attack rate of >x% in x consecutive days, exponential case development, or known diseases with rapid progress and mortality) should be part of a metaphylaxis definition. The concepts of an infectious disease model should be incorporated and represented by the best likelihood of a given infectious disease progressing to an epidemic past a morbidity threshold. For example, a quantitative morbidity threshold which exceeds 10% for 2 to 3 consecutive days was established by Edwards (2010) [39] and Smith et al. (2015) [40] as the best likelihood to apply metaphylaxis to avoid the ‘point of no return’ with mass mortality and animal suffering.

### 3.3. Conditions of Metaphylactic Use per Species

Our study results showed that metaphylactic group treatment was more prominent in pigs and poultry than in other species e.g., cattle. This relates to the production methods of these species, which are often kept in large groups, making it challenging to separate and treat individual animals, as neither the animal nor the caretaker is used to the stress of repeated catching and contention. Furthermore, VMPs formulated for individual treatment are less available for these species. As for cattle, the most indicated group size for metaphylaxis was groups of 0–15 individuals (n = 125/279, 44.8%). In a Belgian study on veal calves, where 88% of antibiotics were administered as metaphylaxis or prophylaxis, the average herd size was 679 calves for dairy, 588 for crossbreeds, and 484 for beef calves [7]. As only 14.3% (n = 40/279) of responding veterinarians indicated treating cattle metaphylactically in herds with more than 100 individuals or more, veterinarians who participated in this survey likely treated adult bovine, which mostly are kept in smaller groups. As metaphylactic treatment in the bovine production is more prominent in veal calves, the distribution of responding bovine veterinarians could partially account for why veterinarians working in practices specialized in cattle were significantly lower metaphylactic group treatment users. This is as well reflected in the significantly lower metaphylactic use in cattle and mixed practices, in which metaphylactic group and therapeutic individual treatment would even out and reach an equal level (Figure 2).

### 3.4. Indications

Responding veterinarians indicated gastrointestinal diseases to be the most common indication for treatment at the weaning stage and fattening/rearing stage. The question frame did not allow for species-specific results, but for pigs, this was in accordance with a study reporting the treatment of gastrointestinal diseases to make up 75% and 60% of indications for prescribed antibiotics over a period of 10 years in Danish weaners and finishers, respectively [41]. In the same study, the second most common indication for both weaners and finishers was respiratory disease, which was also indicated in our survey. This is similar to an EU-wide study that showed that both gastrointestinal together with respiratory indications accounted for more than 60% of antibiotic treatments in pigs [34]. According to our survey, musculoskeletal, locomotor, and neurological diseases only made up a minority of the indications for metaphylactic treatment at the neonatal/hatching phase. This is in contrast to Jensen et al. [41] who reported these indications to make up the majority of prescribed antibiotics during the neonatal phase of piglets. However, the discrepancy can be partially explained by the demographic of responding veterinarians, considering only 21.3% (n = 141) of responding veterinarians worked in practices specialized in pigs. Respiratory diseases made up 40% of indications for metaphylactic treatment of responding veterinarians during the rearing/fattening stage, thereby being the most common indication, alongside gastrointestinal diseases (40%) for treatment during that production phase. An observational study of beef cattle in Northern Italy reported similar results, where antibiotic treatment of respiratory diseases made up the majority of AMU (68%) [42].

The route of administration of different antibiotic classes is influenced inevitably by the availability of VMPs authorized in the respective countries. Within the EU, the availability of VMPs differs greatly, with 296 VMPs including vaccines available in Iceland, compared to 2944 in France [43]. In addition, the administration methods vary between European countries due to the VMP’s licensed differences in preferred administration methods and differences in national action plans [44]. According to the ESVAC report (2021), fluoroquinolones were almost completely administered parentally through injectable products in Sweden, while in Spain around 37% and in Poland around 90% of fluoroquinolones were administered orally [1]. Furthermore, the species most frequently treated with the antibiotic class also influence the formulation of the antibiotic from the pharmaceutical industry perspective. Generally, oral formulations are most often used for group treatments rather than individual treatments, as was reported in an Italian study on antimicrobial use in beef-fattening operations [45] and a similar study on Swiss veal calves [46]. Antibiotics that are primarily administered to poultry and pigs are more likely to be formulated for oral administration, as these species are more likely to be treated as a group through feed or water. However, lower AMU alongside individual administration are both incentivized by national benchmarking systems such as the Danish “yellow card” system, or the Belgian BD100 system, as well as industry-driven quality management approaches [47]. Based on the responses of our survey, third and fourth generation cephalosporins were almost completely administered parentally, while slightly more than half of the macrolides and fluoroquinolones were administered parentally. In a study on antimicrobial usage in farrow-to-finish pig herds in Belgium, Germany, France, and Sweden, similar distributions were found for the administration route of third and fourth generation cephalosporins (88%) and macrolides (61%) [48]. However, in their study, only 4% of fluoroquinolones were administered parentally, a much lower portion than indicated by responding veterinarians in our survey. The data in the study however focused on pigs and had a smaller geographical coverage than this survey, which could explain the discrepancy in the administration route of fluoroquinolones. The relation between route of administration and group or individual treatment can also account for the significantly higher proportion of antibiotics administered per os by high metaphylactic users in poultry compared to low metaphylactic users.

### 3.5. Pathogens Most Commonly Targeted by Metaphylactic Treatment and Treatment Consequences

According to the survey, Enterobacteriaceae were expected to be the most devastating pathogens/disease complex to cause illness and decrease welfare if metaphylaxis would be banned. It was shown before that *E. coli* was regarded as the most common bacterial disease in poultry, resulting in colibacillosis, which refers to a variety of lesions including airsacculitis, septicemia, salpingitis, peritonitis, and omphalitis [49]. In pigs, *E. coli* infections were frequently controlled through metaphylactic antibiotic treatment, but banning metaphylactic treatment was expected to result in economic loss alongside substantial health issues [50]. As seen in our survey, neonatal colibacillosis in pigs was already identified as one of the major therapeutic gaps in France [50]. Vaccination of piglets against *E. coli* strains was shown as an effective alternative to control post-weaning diarrhea at farm level [51,52]. Though both an oral live vaccine and an intramuscular toxoid vaccine are available, their spectrum is limited to certain *E. coli* strains (*E. coli* F4/F18 and STx2e producing *E. coli*, resp.). However, a significant association between increased vaccination of piglets against *E. coli* and reduction of colistin use was seen in Estonia [52]. Novel technologies such as subunit vaccines could be used as a single vaccine across the farrowing, suckling, and weaning program to protect against pathogenic *E. coli* [53]. The impact of these pathogens is accelerated by the availability shortages of other appropriated alternatives such as non-antimicrobial feed additives [54].

Responding veterinarians indicated increased mortality, increased morbidity, and economic loss to be the three most significant health consequences if metaphylaxis were banned. This confirmed earlier findings, as metaphylaxis was shown to be an effective tool used to maintain herd health and decrease morbidity and mortality in beef cattle [37]. Banning metaphylaxis without implementing changes in husbandry systems or other alternative measures would result in increased mortality, morbidity and, therefore, economic loss [55]. Practical and management issues were mostly indicated by poultry practitioners. With metaphylaxis as the only feasible manner to treat commercially held poultry, banning metaphylaxis in the current poultry husbandry systems would be very challenging. While the treatment frequency of poultry in certain countries can and shall decrease, this should preferably be done by creating well-defined treatment guidelines on a national level for practitioners which allow in a targeted way for increased clarity on morbidity thresholds per disease and on the AMR profile of identified pathogens, which should be determined before poultry flocks receive treatment.

The most indicated alternatives to antibiotics as seen in the survey were vaccination, biosecurity including hygiene measures, and improved herd health management. These are all strategies that have shown to be effective in decreasing pathogen exposure, thereby reducing disease incidence and the need for antibiotic treatment. However, none of the alternative measures can completely replace antibiotic group treatment in an epidemic disease development. With the EU regulation 2019/6 laying the legal groundwork for autogenous vaccines, these could be implemented more often as a vital tool to combat bacterial infections and reduce AMU [5]. While promising developments have been made, especially in the case of vaccines, alternatives must be combined in a holistic hurdle approach [56]. A hurdle system features a range of synergistic measures, including vaccinations and biosecurity, with each hurdle playing an essential role to decrease firstly, the risk of exposure to pathogens, and secondly, the spread of pathogens. It was initially implemented on food preservation [57]. Measures such as vaccination protocols and internal and external biosecurity, including hygiene measures, are included in the BioCheck.UGent system, which scores the biosecurity of livestock and poultry farms to assess the risk of pathogen introduction and spread [58]. Using the BioCheck.UGent tool to score and assess the biosecurity practices of 58 Irish farrow to finish pig farms, biosecurity practices accounted for 8% and 23% of piglet and finisher mortality, respectively [59]. Furthermore, when the BioCheck.UGent tool was used to score 30 Dutch and Belgian broiler farms and subsequently educate farmers on improving their biosecurity and on antimicrobial stewardship, a 6% increase in biosecurity and 7% reduction of AMU, without negative effects on production parameters, was reported [60]. On the European level, the effective implementation of different alternative measures in pig production, such as improvement of biosecurity, vaccination, improved feeding, and health care, has resulted in a significant reduction in AMU [61]. Adequate hygiene measures must be employed to alleviate underlying reasons of reoccurrence of avian pathogenic *E. coli* on poultry farms such as biofilm formation [62,63]. However, the implementation of alternatives in a hurdle system must be feasible for farmers, as they often require financial investments. Interviewed livestock veterinarians in the Netherlands indicated economic considerations as a major factor in the decision on whether or not to opt for alternative measures [64]. Other factors such as climate and accessibility also influence the efficacy of alternative measures, as well as the possibility and motivation of farmers to implement them. Digitalization, including precision livestock farming (PLF) tools to monitor herd health, offer great potential, but currently have drawbacks in their availability and scalability [57,65]. While being a promising step for the livestock industry, scalable commercialization is necessary to offer a consistent and economically viable service to farmers for PLF to be implemented at farm level [66].

### 3.6. Limitations of the Study

The linguistic accessibility and geographical coverage of the survey resulted in a sufficient sample size, supported by multi-language questionnaires. However, even well-translated surveys can be biased by cultural issues. Our main considerations were cross-cultural equivalence of scales, and whether respondents could be biased toward answering questions in ways that are socially acceptable. The snowball sampling strategy made it challenging to determine the overall response rate, sampling error, or generalize inferences solely based on a purely voluntary call for participation of the obtained questionnaire responses. Furthermore, most of the responses were received from four countries with above-average antibiotic sales [1] and six countries were without any responses, which affected the representativeness and limits the extrapolation to a full European view. As the use of antibiotics and the prevalence of metaphylactic and group treatments differ between EU member states, the geographical distribution of responses could influence the results of the survey, leading to a bias. In addition, FVE informed respondents of the survey about its concerns of a wide ban of metaphylaxis, which may result in high morbidity, mortality, and devastating production losses. This has the potential to led to contextual bias as the survey relied on voluntary responses from practitioners. A certain element of bias of the study was that responding veterinarians could be those more conscious of judicious antibiotic use and consequently, administrating or prescribing antibiotics more prudently. The request for a follow-up on the results of the survey from 368 of the 662 responding veterinarians represents a large interest from practitioners surrounding the subject of metaphylactic antibiotic treatments. Despite these limitations, the results of the survey give valuable insight into the way in which livestock veterinarians apply metaphylactic treatment in Europe.

## 4. Materials and Methods

### 4.1. Metaphylaxis Survey

The survey was developed by FVE together with four European veterinary field experts on poultry, porcine, and bovine health to ensure content validity of the survey. The STROBE (Strengthening the Reporting of Observational Studies in Epidemiology) guideline for cross-sectional studies [67] and the Checklist for Reporting Results of Internet E-Surveys (CHERRIES) [68] were used for reporting (Supplementary Materials Tables S3 and S4). Psychometric testing of the survey was not performed. Prior to distribution, formal testing of the questionnaire was carried out by 18 representative veterinarians from all sectors and various European countries to identify potential interpretation difficulties, and any unclear questions were adjusted. Targeted e-mails with an open link to the questionnaire on Google Forms were sent to the 51 national veterinary associations as members of FVE, requesting to forward the survey to their respective individual members. The survey form was available in nine languages and included nine questions. Participants were given appropriate project information, including content, sponsorship, and purpose. Participation and each question were voluntary and not remunerated. The online survey was accessible between 23 December 2021 and 15 February 2022 with one reminder on 30 January 2021. Questions covered demographics, the absolute and percentual use, decision-making process, and most common patterns (group size, production stage, disease, antibiotics class and administration route) of metaphylactic group treatment per species. Responses to Q5 are not shown in this manuscript. Responses to open questions (Q8 and 8d) were standardized and categorized for harmonization purposes. Multiple answers were accepted to account for veterinarians working with various species, resulting in varying group totals. With respect to the most common patterns, veterinarians were asked to specifically refer to the last 3 months in their practice to minimize social desirability/response bias (i.e., describing best or generally applied practices rather than the actual practices) and recall bias, given the assumed short period of time between treatments and survey participation. The “(sub)species-diseases–pathogen” combinations with the most devastating effect and their potential consequences were recorded per species. The questionnaire ended with a list of most effective alternative measures to prevent and/or to treat diseases other than metaphylactically with antibiotics that must become available to implement a consistent change (Supplementary Table S2). All responses were editable by the participants until the survey was closed.

### 4.2. Data Handling and Statistical Analysis

Data were collected anonymously, unless participants wished to provide an email address of their own accord and with informed consent. Any potential contact details or names mentioned by participants during the research were anonymized after transcription. Incomplete or duplicate responses based on time stamps were removed and the first entry was kept for analysis. After cleaning, data on terrestrial livestock and avian species were tabulated, processed in Microsoft^®^ Excel, and organized by metaphylactic use (group 1: ≤74%, group 2: ≥75%) and type of practice (mixed, specialized in pigs, specialized in poultry, specialized in cattle, and other (rabbits and small ruminants)). Chi-square (χ2) tests and a logistic regression model were used to evaluate differences between the impact of the independent variables on the use of metaphylaxis. Calculations were performed in RStudio (package stats) and GraphPad software (San Diego, CA, USA). A *p*-value of ≤0.05 was considered significant.

## 5. Conclusions

The new EU veterinary medicines Regulation (EC) 2019/6 stipulates that antimicrobials as metaphylaxis should only be used where the risk of spreading a contagious bacterial disease is high and no other appropriate alternatives are available. Veterinary professional associations strongly advocate the principles of antimicrobial stewardship and responsible use, yet the survey results indicated that a ban of metaphylactic group treatment will likely result in high morbidity and mortality, mostly due to infections with Gram-negative bacteria such as Enterobacteriaceae. Therefore, specific indications require inevitably whole-group treatment of livestock and poultry in an epidemic disease development to effectively maintain animal health and welfare. The spread of disease was a major driver for the initiation of metaphylaxis, acknowledging that the control of a disease epidemic should be the true purpose of antibiotic metaphylaxis. Further injudicious restriction in the availability of veterinary antibiotics intended for flock, group, or herd medication may result in a practical ban of effective treatment by metaphylaxis in animal husbandry. More research is needed to implement appropriate alternatives in a holistic hurdle approach such as improved farming conditions, biosecurity measures in between production cycles, and vaccination as decreased need to use antibiotics will not be achieved by a single alternative measure. Other alternatives such as pro- and prebiotic feed additives would also be beneficial to be included in the hurdle system. In addition, active support will be necessary for the development and application of targeted national decision treatment guidelines for practitioners taking into account husbandry systems, rearing conditions, and specialization, which promote the understanding of drivers and include criteria for initiation of metaphylaxis in livestock.

## Figures and Tables

**Table 1:** Basis of decision making in absolute numbers (Q7 with n = 1158 survey responses, multiple answers possible) and percent for each decision option per practice type to initiate metaphylactic treatment.

	Decision Making-Basis	Depending on the Severity of the Symptoms and the Suspected Agent/ Condition Involved	Known Disease Which Spreads Quickly	Diagnosis of Further Laboratory Testing/Microbiology/ In Vitro Sensitivity Testing
Type of Practice				
Mixed practice		39.3%	35.3%	25.4%
		(n = 161)	(n = 145)	(n = 104)
Practice specialized in pigs		38.7%	37.4%	23.9%
		(n = 102)	(n = 99)	(n = 63)
Practice specialized in cattle		36.6%	40.5%	22.9%
		(n = 96)	(n = 106)	(n = 60)
Practice specialized in poultry, incl. chicken and turkeys		36.7%	28.2%	35.1%
		(n = 70)	(n = 54)	(n = 67)
Other	Cuniculture	31.2%	31.3%	37.5%
		(n = 5)	(n = 5)	(n = 6)
	Practice specialized in sheep and goats	40%	33.3%	26.7%
		(n = 6)	(n = 5)	(n = 4)
Total		38%	35.8%	26.2%
		(n = 440)	(n = 414)	(n = 304)

**Table 2:** Most significant health and welfare consequences for the most frequent model of metaphylactic treatment outlined per practice type if metaphylaxis were prohibited (Q8d with 998 responses, multiple answer possible).

	Practice Type	Mixed Practice	Cattle Practice	Pig Practice	Poultry Practice	Sheep/Goat Practice	Cuniculture Practice	Total
Consequences								
Increased mortality		42.2%	43.1%	46.0%	35.9%	57.1%	30.8%	42.2%
		(n = 129)	(n = 110)	(n = 104)	(n = 66)	(n = 8)	(n = 4)	(n = 421)
Increased morbidity		21.9%	20.8%	22.6%	13.6%	14.3%	23.1%	20.1%
		(n = 67)	(n = 53)	(n = 51)	(n = 25)	(n = 2)	(n = 3)	(n = 201)
Decreased production and economic loss		14.0%	12.9%	14.2%	11.4%	14.3%	23.1%	13.4%
		(n = 43)	(n = 33)	(n = 32)	(n = 21)	(n = 2)	(n = 3)	(n = 134)
Lower welfare		8.2%	7.5%	8.4%	18.5%	7.1%	15.4%	10.0%
		(n = 25)	(n = 19)	(n = 19)	(n = 34)	(n = 1)	(n = 2)	(n = 100)
Increased antibiotic treatment		5.9%	6.3%	4.9%	3.8%	0.00%	0.00%	5.2%
		(n = 18)	(n = 16)	(n = 11)	(n = 7)	(n = 0)	(n = 0)	(n = 52)
Increased chronicity		4.3%	7.0%	1.8%	3.3%	0.00%	0.00%	4.1%
		(n = 13)	(n = 18)	(n = 4)	(n = 6)	(n = 0)	(n = 0)	(n = 41)
Practical/Management issues		1.0%	0.8%	1.3%	10.3%	0.00%	7.7%	2.8%
		(n = 3)	(n = 2)	(n = 3)	(n = 19)	(n = 0)	(n = 1)	(n = 28)
None		2.3%	1.6%	0.5%	1.1%	7.1%	0.00%	1.5%
		(n = 7)	(n = 4)	(n = 1)	(n = 2)	(n = 1)	(n = 0)	(n = 15)
Public health risk		0.3%	0.00%	0.4%	2.2%	0.00%	0.00%	0.6%
		(n = 1)	(n = 0)	(n = 1)	(n = 4)	(n = 0)	(n = 0)	(n = 6)

## Data Availability

The datasets analyzed for this study are available on request from the corresponding author, N.D.B. The raw data are not publicly available due to their containing information that could compromise the privacy of research participants.

## Supplementary Materials

The following supporting information can be downloaded at: https://www.mdpi.com/article/10.3390/antibiotics11081046/s1, Table S1: Demographic distribution of survey responses; Table S2: EN-Metaphylaxis in livestock and poultry survey form; Table S3: STROBE statement; Table S4: Checklist for Reporting Results of Internet E-Surveys (CHERRIES).
