# Supplementary material for: Real-World Data on Antibiotic Group Treatment in European Livestock: Drivers, Conditions, and Alternatives
Source: Antibiotics (Basel). 2022 Aug 3;11(8):1046. doi: 10.3390/antibiotics11081046 (PMC9404736; doi:10.3390/antibiotics11081046)
Supplement: Supplementary file 1 [file antibiotics-11-01046-s001.zip › Table S1. Demographic distribution of survey responses_R1 .pdf]

Table S1. Demographic features of responding veterinarians

Countries, practice type and size \ Experience	<5 years	> 25 years	16-25 years	6-15 years	Total
**Armenia**			**1**		**1**
**Practice specialised in cattle**			**1**		**1**
Large practice			1		1
**Austria**			**1**		**1**
**Practice specialised in poultry, incl. chicken and turkeys**			**1**		**1**
Medium practice			1		1
**Belgium**		**2**			**2**
**Mixed practice**		**1**			**1**
Medium practice		1			1
**Practice specialised in poultry, incl. chicken and turkeys**		**1**			**1**
Large practice		1			1
**Bulgaria**	**1**	**2**	**1**	**2**	**6**
**Mixed practice**	**1**	**1**		**1**	**3**
Large practice	1				1
Very small practice		1		1	2
**Practice specialised in cattle**		**1**		**1**	**2**
Very small practice				1	1
(blank)		1			1
**Practice specialised in poultry, incl. chicken and turkeys**			**1**		**1**
Large practice			1		1
**Croatia**			**1**		**1**
**Mixed practice**			**1**		**1**
Large practice			1		1
**Czech Republic**		**3**			**3**
**Mixed practice**		**1**			**1**
Very small practice		1			1
**Practice specialised in pigs**		**2**			**2**
Very small practice		2			2
**Denmark**	**1**	**5**			**6**

**Mixed practice**		**1**			**1**
Small practice		1			1
**Practice specialised in pigs**	**1**	**4**			**5**
Large practice		2			2
Small practice	1	1			2
Very small practice		1			1
**Finland**		**1**	**1**	**3**	**5**
**Mixed practice**			**1**	**3**	**4**
Very small practice			1	3	4
**Practice specialised in poultry, incl. chicken and turkeys**		**1**			**1**
Medium practice		1			1
**France**	**6**	**42**	**28**	**32**	**108**
**Cuniculture**		**1**			**1**
Large practice		1			1
**Mixed practice**	**5**	**13**	**12**	**14**	**44**
Large practice	4	4	6	1	15
Medium practice		3	5	6	14
Small practice	1	3		5	9
Very small practice		3	1	2	6
**Practice specialised in cattle**		**8**	**4**	**6**	**18**
Large practice			2	3	5
Medium practice		4	1	1	6
Small practice		3	1	2	6
Very small practice		1			1
**Practice specialised in pigs**	**1**	**15**	**4**	**4**	**24**
Large practice	1	8	2	2	13
Medium practice		6	1	1	8
Small practice			1		1
Very small practice		1		1	2
**Practice specialised in poultry, incl. chicken and turkeys**		**5**	**7**	**7**	**19**
Large practice		1	4	4	9
Medium practice		2	1	2	5
Small practice		1	1	1	3
Very small practice		1	1		2

**Practice specialised in sheep and goats**			**1**	**1**	**2**
Very small practice			1	1	2
**Germany**	**5**	**26**	**13**	**17**	**61**
**Mixed practice**	**2**	**15**	**8**	**4**	**29**
Large practice	1	1			2
Medium practice		1	2		3
Small practice	1	7	1		9
Very small practice		6	5	4	15
**Practice specialised in cattle**	**1**	**3**		**1**	**5**
Small practice	1				1
Very small practice		3		1	4
**Practice specialised in pigs**	**1**	**4**		**4**	**9**
Large practice	1	1		1	3
Medium practice		1		1	2
Small practice		1			1
Very small practice		1		2	3
**Practice specialised in poultry, incl. chicken and turkeys**	**1**	**4**	**5**	**8**	**18**
Large practice			1	6	7
Medium practice	1	1	2	1	5
Small practice		2	1		3
Very small practice		1	1	1	3
**Greece**	**2**	**11**	**6**	**7**	**26**
**Mixed practice**	**2**	**8**	**3**	**4**	**17**
Very small practice	2	8	3	4	17
**Practice specialised in cattle**			**2**	**1**	**3**
Large practice			1		1
Very small practice			1	1	2
**Practice specialised in pigs**				**1**	**1**
Very small practice				1	1
**Practice specialised in poultry, incl. chicken and turkeys**		**3**			**3**
Small practice		1			1
Very small practice		2			2
**Practice specialised in sheep and goats**			**1**	**1**	**2**
Small practice			1		1

Very small practice				1	1
**Hungary**	**1**	**28**	**9**	**8**	**46**
**Mixed practice**	**1**	**21**	**8**	**5**	**35**
Very small practice	1	20	8	5	34
(blank)		1			1
**Practice specialised in cattle**		**1**			**1**
Very small practice		1			1
**Practice specialised in pigs**		**3**	**1**	**2**	**6**
Small practice		1			1
Very small practice		2	1	2	5
**Practice specialised in poultry, incl. chicken and turkeys**		**3**		**1**	**4**
Very small practice		3		1	4
**Ireland**				**2**	**2**
**Mixed practice**				**2**	**2**
Very small practice				2	2
**Italy**	**2**	**13**	**7**	**2**	**24**
**Mixed practice**		**3**			**3**
Large practice		2			2
Very small practice		1			1
**Practice specialised in cattle**	**1**	**5**	**2**	**2**	**10**
Small practice				1	1
Very small practice	1	4	2	1	8
(blank)		1			1
**Practice specialised in pigs**	**1**	**3**	**3**		**7**
Large practice			1		1
Small practice	1				1
Very small practice		3	2		5
**Practice specialised in poultry, incl. chicken and turkeys**		**2**	**1**		**3**
Medium practice		1			1
Very small practice		1	1		2
**Practice specialised in sheep and goats**			**1**		**1**
(blank)			1		1
**Latvia**		**1**			**1**
**Mixed practice**		**1**			**1**

Very small practice		1			1
**Luxembourg**		**1**		**2**	**3**
**Mixed practice**		**1**		**2**	**3**
Small practice				2	2
Very small practice		1			1
**Malta**				**1**	**1**
**Mixed practice**				**1**	**1**
Very small practice				1	1
**Netherlands**	**2**	**16**	**12**	**12**	**42**
**Mixed practice**		**6**	**4**	**3**	**13**
Large practice		4	2	3	9
Medium practice			1		1
Small practice		1			1
Very small practice		1	1		2
**Practice specialised in cattle**		**2**	**1**		**3**
Large practice		1	1		2
Medium practice		1			1
**Practice specialised in pigs**		**6**	**4**	**6**	**16**
Large practice		3	3	2	8
Medium practice		1		3	4
Small practice		1	1	1	3
Very small practice		1			1
**Practice specialised in poultry, incl. chicken and turkeys**	**2**	**2**	**3**	**3**	**10**
Large practice	1	1	1	3	6
Medium practice			1		1
Small practice	1	1	1		3
**Norway**		**1**			**1**
**Mixed practice**		**1**			**1**
Medium practice		1			1
**Other**	**2**	**10**	**6**	**3**	**21**
**Mixed practice**		**4**	**4**	**1**	**9**
Small practice		1			1
Very small practice		3	4	1	8
**Practice specialised in cattle**		**3**	**2**		**5**

Large practice			1		1
Medium practice		1			1
Small practice		1			1
Very small practice		1	1		2
**Practice specialised in pigs**	**1**	**3**		**1**	**5**
Small practice	1	1			2
Very small practice		2		1	3
**Practice specialised in poultry, incl. chicken and turkeys**	**1**			**1**	**2**
Small practice				1	1
(blank)	1				1
**Poland**	**4**	**15**	**8**	**8**	**35**
**Mixed practice**	**1**	**10**	**5**	**2**	**18**
Large practice		1			1
Very small practice	1	9	5	2	17
**Practice specialised in cattle**		**1**	**1**	**3**	**5**
Small practice		1			1
Very small practice			1	3	4
**Practice specialised in pigs**	**1**	**2**		**1**	**4**
Small practice	1			1	2
Very small practice		2			2
**Practice specialised in poultry, incl. chicken and turkeys**	**2**	**2**	**2**	**2**	**8**
Medium practice	1				1
Small practice			1	1	2
Very small practice	1	2	1	1	5
**Portugal**		**1**			**1**
**Practice specialised in poultry, incl. chicken and turkeys**		**1**			**1**
Very small practice		1			1
**Romania**			**1**	**2**	**3**
**Mixed practice**				**1**	**1**
Very small practice				1	1
**Practice specialised in pigs**			**1**		**1**
Very small practice			1		1
**Practice specialised in poultry, incl. chicken and turkeys**				**1**	**1**
Small practice				1	1

**Slovakia**		**1**	**1**		**2**
**Mixed practice**		**1**			**1**
Very small practice		1			1
**Practice specialised in pigs**			**1**		**1**
Very small practice			1		1
**Slovenia**	**1**	**3**	**2**	**1**	**7**
**Mixed practice**		**3**	**1**	**1**	**5**
Small practice		1	1		2
Very small practice		2		1	3
**Practice specialised in cattle**	**1**		**1**		**2**
Very small practice	1		1		2
**Spain**	**22**	**82**	**75**	**48**	**227**
**Cuniculture**		**5**	**2**		**7**
Large practice		1			1
Medium practice		1			1
Small practice		2	2		4
Very small practice		1			1
**Mixed practice**	**3**	**17**	**12**	**6**	**38**
Large practice		6	1	2	9
Medium practice		1		1	2
Small practice		4	1		5
Very small practice	3	6	10	3	22
**Practice specialised in cattle**	**13**	**34**	**38**	**23**	**108**
Large practice	1	6	7	5	19
Medium practice	1	2	6	1	10
Small practice	3	5	5	3	16
Very small practice	8	20	20	13	61
(blank)		1		1	2
**Practice specialised in pigs**	**5**	**23**	**16**	**13**	**57**
Large practice	1	8	2	5	16
Medium practice		1	1	2	4
Small practice		3	3	3	9
Very small practice	4	11	10	3	28
**Practice specialised in poultry, incl. chicken and turkeys**		**3**	**4**	**5**	**12**

Large practice		1	1	1	3
Small practice			2		2
Very small practice		2	1	4	7
**Practice specialised in sheep and goats**	**1**		**3**	**1**	**5**
Large practice				1	1
Medium practice			1		1
Very small practice	1		2		3
**Sweden**	**3**	**2**	**4**	**6**	**15**
**Mixed practice**	**3**		**1**	**5**	**9**
Large practice				1	1
Medium practice	2			3	5
Small practice	1		1	1	3
**Practice specialised in cattle**		**1**	**1**	**1**	**3**
Large practice		1			1
Small practice			1		1
Very small practice				1	1
**Practice specialised in poultry, incl. chicken and turkeys**		**1**	**2**		**3**
Very small practice		1	2		3
**Switzerland**		**2**		**2**	**4**
**Practice specialised in pigs**		**1**			**1**
Small practice		1			1
**Practice specialised in poultry, incl. chicken and turkeys**		**1**		**2**	**3**
Very small practice		1		2	3
**United Kingdom**	**2**	**3**	**1**	**1**	**7**
**Practice specialised in pigs**		**1**	**1**		**2**
Large practice		1	1		2
**Practice specialised in poultry, incl. chicken and turkeys**	**2**	**2**		**1**	**5**
Large practice	1			1	2
Small practice	1				1
Very small practice		2			2
**Total**	**54**	**271**	**178**	**159**	**662**
