# Supplementary material for: Real-World Data on Antibiotic Group Treatment in European Livestock: Drivers, Conditions, and Alternatives
Source: Antibiotics (Basel). 2022 Aug 3;11(8):1046. doi: 10.3390/antibiotics11081046 (PMC9404736; doi:10.3390/antibiotics11081046)
Supplement: Supplementary file 1 [file antibiotics-11-01046-s001.zip › Table S2. EN - Metaphylaxis in livestock and poultry survey form_R1.pdf]

Table S2. FVE Survey on Metaphylaxis in Livestock and Poultry

**Title**	**FVE Survey on Metaphylaxis in Livestock and Poultry**
**Introduction**	Major efforts have been made by the veterinary profession to reduce the need for antimicrobial use in farmed animals. However, animals can become sick even under the best rearing conditions and may need to be treated with antimicrobials. Metaphylaxis is defined in the Regulation 6/2019 by "the administration of a medicinal product to a group of animals after a diagnosis of clinical disease in part of the group has been established, with the aim of treating the clinically sick animals and controlling the spread of the disease to animals in close contact and at risk and which may already be subclinically infected" and this definition is supported by EMA.<br><br>However, a wide ban of metaphylaxis may result in high morbidity, mortality and devastating production losses. This new Regulation (EC) 2019/6 stipulates that antimicrobials as metaphylaxis should only be used where the risk of spreading a contagious bacterial disease is high and no other appropriate alternatives are available. Colistin, often used for metaphylaxis, has been categorised by WHO as critically important antibiotic of highest priority, spotlighting its use in veterinary medicine. FVE strongly advocates the principles of antimicrobial stewardship and responsible use but believes that some indications require inevitably whole group treatment of farmed animals to effectively maintain animal health and welfare. Further injudicious restriction in the availability of veterinary antibiotics intended for flock, group or herd medication may result in a practical ban of effective treatment by metaphylaxis in animal husbandry.<br><br>In this short survey, we would like to gather field experience in regard of metaphylaxis with a special emphasis on colistin use. We aim to gain information on the most frequent indications for metaphylaxis group treatment, to investigate possible alternative strategies, and to identify lessons learned. Please indicate below how and why you treated food-producing animals metaphylactically. If you would like to receive a summary of the survey outcome, please fill in your email address below. We will contact we as soon as the report is ready.<br><br>Data will be handled anonymously and in line with the EU GDPR rules.<br><br>For questions please contact Wiebke Jansen, FVE (info@fve.org)

Table S2. FVE Survey on Metaphylaxis in Livestock and Poultry

Question	Answer type	Answer options				
1. In which country do you work as a veterinary practitioner?	Multiple Choice	Albania	Armenia	Austria	Belgium	
		Bosnia/Herzegovina	Bulgaria	Croatia	Cyprus	
		Czech Republic	Denmark	Estonia	Finland	
		France	Germany	Greece	Hungary	
		Iceland	Ireland	Italy	Latvia	
		Lithuania	Luxembourg	Malta	Montenegro	
		Netherlands	North Macedonia	Norway	Poland	
		Portugal	Romania	Russia	Serbia	
		Slovakia	Slovenia	Spain	Sweden	
		Switzerland	Ukraine	United Kingdom	Other	
2. How many years of experience do you have as a veterinary practitioner?	Multiple Choice	<5 years	6-15 years	16-25 years	>25 years	
3. In which type of practice do you work?	Multiple Choice	Mixed practice	Practice specialised in pigs	Practice specialised in poultry, incl. chicken and turkeys	Practice specialised in cattle	Other:
4. How many livestock veterinarians work in your practice?	Multiple Choice	1-3	4-6	7-9	>10	
5. Is metaphylaxis required as a treatment option in your routine work? (Only answer for species that you treat)	Multiple Choice Grid	Never	Rarely	Occasionally	Often	
	Cattle incl. calves					
	Poultry					
	Pigs incl. piglets					
	Other					

Table S2. FVE Survey on Metaphylaxis in Livestock and Poultry

6. Which percentage of your treatments is represented by metaphylaxis or group treatment? (Only answer for the species that you treat)	Multiple Choice Grid	<25%	>25%	>50%	>75%	>90%
	Cattle incl. calves					
	Poultry					
	Pigs incl. piglets					
	Other					

7. When do you take the decision to apply metaphylactic treatment?	Multiple Choice	Depending on the severity of signs and the suspected agent/condition involved	Diagnosis of further laboratory testing/microbiology/in vitro sensitivity testing	Known disease which spreads quickly

8. Based on your experience, which "(sub)species - diseases – pathogen" combination would have the most devastating effect regarding animal health and welfare (morbidity, mortality, production loss, ..) if metaphylaxis would be banned? You can give up to 5 combinations. Example : Broiler chicken - septicemia - *E. coli*	Long Paragraph	

8a. Please indicate here the one most frequent pattern for metaphylactic treatment that you apply in your practice by ticking the corresponding boxes - species and group size	Multiple Choice Grid	Cattle	Pigs	Poultry	Other farmed animals
	0-15 individuals				
	16-30 individuals				

Table S2. FVE Survey on Metaphylaxis in Livestock and Poultry

	>30 individuals						
	>100 individuals						
	>1000 individuals						
8b. Please indicate here the one most frequent pattern for metaphylactic treatment that you apply in your practice by ticking the corresponding boxes - production stage and disease	Multiple Choice Grid	Septicaemia	Respiratory diseases	Gastro-intestinal diseases	Mastitis/ metritis	Loco-motor diseases	Neuro-logical diseases
	Neonatal/ at hatch						
	At weaning						
	After transport/ newly grouped						
	Fattening/ rearing						
	Breeding/ postpartum						
	In lay/ lactation						
8c. Please indicate here the one most frequent pattern for metaphylactic treatment that you apply in your practice by ticking the corresponding boxes - antibiotic and administration route	Multiple Choice Grid	Per os- premixed feed	Per os- feed top dressing	Per os- drinking water	Parenteral- sub-cutaneous	Parenteral- intra-muscular	Parenteral intrave

Table S2. FVE Survey on Metaphylaxis in Livestock and Poultry

							no us
	(Fluoro)-quinolones						
	Colistin						
	Macrolides						
	3G or 4G cephalon-sporins						
	Amino-glycosides						
	Penicillins without beta-lactam inhibitors						
	Penicillins with beta-lactcam inhibitors						
8d. Based on your examples, which would be the most significant health and welfare consequences for the condition that you indicated above if metaphylaxis would be banned?	Long paragraph						
9. Please indicate the three most effective alternative measures to prevent and/or to treat diseases other than metaphylactically with antibiotics (and particularly colistin) that must become available to implement a consistent change?	Multiple Choice	Vaccination		Other/new licensed antibiotics		Other non-antibiotic treatments (e.g. NSAIDs)	
		Improved housing		Improved hygiene measures		Improved feeding	

Table S2. FVE Survey on Metaphylaxis in Livestock and Poultry

		Improved herd health management	Structural changes e.g. later weaning	Other:
If you have additional information on your use of metaphylaxis, please share them here:	Short paragraph			
If you would like to receive a summary of the survey outcome, please fill in your email address here. We will contact we as soon as the report is ready.	Short paragraph			
